# Chronic kidney disease is associated with adverse outcomes among elderly patients taking clopidogrel after hospitalization for acute coronary syndrome

**DOI:** 10.1186/1471-2369-14-107

**Published:** 2013-05-20

**Authors:** Michael J Fischer, P Michael Ho, Kelly McDermott, Elliott Lowy, Chirag R Parikh

**Affiliations:** 1 Jesse Brown VAMC/University of Illinois Medical Center, Chicago, IL, USA; 2 Center for the Management of Complex Chronic Care, Hines VA Hospital, 5000 S. 5th Avenue (151H), Hines, IL, USA; 3 Denver VA Medical Center, Denver, CO, USA; 4 Osher Center for Integrative Medicine, University of California, San Francisco, CA, USA; 5 VA Puget Sound Healthcare System/University of Washington, Seattle, WA, USA; 6 Clinical Epidemiology Research Center, West Haven VA; Program of Applied Translational Research, Department of Medicine, Yale University, New Haven, CT, USA

**Keywords:** Kidney disease, Myocardial infarction, Hospitalization, Bleeding

## Abstract

**Background:**

Chronic kidney disease (CKD) is associated with worse outcomes among patients with acute coronary syndrome (ACS). Less is known about the impact of CKD on longitudinal outcomes among clopidogrel treated patients following ACS.

**Methods:**

Using a retrospective cohort design, we identified patients hospitalized with ACS between 10/1/2005 and 1/10/10 at Department of Veterans Affairs (VA) facilities and who were discharged on clopidogrel. Using outpatient serum creatinine values, estimated glomerular filtration rate [eGFR (1.73 ml/min/m^2^)] was calculated using the CKD-EPI equation. The association between eGFR and mortality, hospitalization for acute myocardial infarction (AMI), and major bleeding were examined using Cox proportional hazards models.

**Results:**

Among 7413 patients hospitalized with ACS and discharged taking clopidogrel, 34.5% had eGFR 30–60 and 11.6% had eGFR < 30. During 1-year follow-up after hospital discharge, 10% of the cohort died, 18% were hospitalized for AMI, and 4% had a major bleeding event. Compared to those with eGFR > =60, individuals with eGFR 30–60 (HR 1.45; 95% CI: 1.18-1.76) and < 30 (HR 2.48; 95% CI: 1.97-3.13) had a significantly higher risk of death. A progressive increased risk of AMI hospitalization was associated with declining eGFR: HR 1.20; 95% CI: 1.04-1.37 for eGFR 30–60 and HR 1.47; 95% CI: 1.22-1.78 for eGFR < 30. eGFR < 30 was independently associated with over a 2-fold increased risk in major bleeding (HR 2.09; 95% CI: 1.40-3.12) compared with eGFR > = 60.

**Conclusion:**

Lower levels of kidney function were associated with higher rates of death, AMI hospitalization, and major bleeding among patients taking clopidogrel after hospitalization for ACS.

## Background

Approximately 30% to 50% of patients with acute coronary syndrome (ACS) have evidence of chronic kidney disease (CKD) [1,2]. Patients with CKD have worse outcomes (i.e., recurrent adverse cardiac events, longer hospitalization, and increased mortality) following ACS regardless of treatment strategy, percutaneous coronary intervention (PCI) or medical therapy [3-14]. While clopidogrel has been shown to reduce vascular related mortality and cardiovascular (CV) events in high-risk populations by inhibiting intravascular thrombosis [15,16], post-hoc subgroup analyses of two clinical trials demonstrated that clopidogrel has a less beneficial effect on reducing the risk of cardiovascular events for adults with CKD [17,18].

A number of reasons may explain the worse outcomes associated with CKD, including more complex coronary anatomy, a greater atherosclerotic burden, an underlying prothrombotic state, and decrease platelet responsiveness to antiplatelet agents in the setting of CKD [19-25]. A few observational studies examining outcomes among representative groups of patients discharged on clopidogrel following PCI found that the presence of CKD was associated with adverse ischemic events and bleeding [26-28]. However, these studies were limited because they did not account for differences in clopidogrel treatment duration, did not include patients treated medically without PCI for ACS, or had a paucity of patients with severe CKD, all of which have important implications for outcomes [26-28].

In order to improve our understanding of the impact of CKD in adults treated with clopidogrel after ACS, our objective was to examine the relationship between levels of kidney function and a range of outcomes, namely mortality, recurrent myocardial infarction, and bleeding, in a large national cohort of elderly patients hospitalized for ACS. Specifically, we address limitations of the prior literature because our cohort includes patients with a broad range of kidney function, even those with severe CKD, patient treated both medically and with PCI for ACS, and data on clopidogrel treatment duration during follow-up. We hypothesized that lower levels of kidney function would be associated with increased rates of death, hospitalization, and bleeding in this clopidogrel-treated cohort following ACS.

## Methods

### Design and study sample

Using a retrospective cohort study design, data for this study were collected as part of the Department of Veterans Affairs (VA) Veterans Health Administration Cardiac Care Follow-up Clinical Study, which has been described in detail elsewhere [29,30]. Briefly, the records of all patients with ACS, as defined by acute myocardial infarction (AMI) and a random sample of all patients with unstable angina (UA), discharged from a VA hospital were abstracted as part of a national VA cardiac care initiative. All patients with *International Classification of Diseases, Ninth Revision, Clinical Modification* (ICD-9CM) diagnosis codes 410.xx (acute myocardial infarction) and 411.xx (other acute and subacute forms of ischemic heart disease) were identified from the VA Patient Treatment File, and their records were manually abstracted by trained abstractors using standard reporting forms. Additional details of the study methods have been published [31].

Based on the above criteria, the study cohort included 22,948 patients who presented to a VA facility between October 1, 2005, and January 10, 2010 with an ACS. We included all patients who were admitted and hospitalized, survived to hospital discharge, and were prescribed clopidogrel at time of discharge. We excluded 8,757 patients who received palliative care, had decisions not to treat, or received same day discharge or non-routine discharges (i.e., transfers), 4,952 were excluded who did not receive clopidogrel at discharge, and an additional 1826 were excluded who lacked an eGFR value. Hence, our final analytic study cohort was 7,413.

### Variables and data sources

Clopidogrel use was assessed using the Veterans Health Administration Pharmacy Benefits Management database, which records the date dispensed and the number of days supplied for each dispensed medication. Duration of clopidogrel therapy was calculated from the day of hospital discharge to the last clopidogrel refill date plus the number of days supplied for that last refill [32,33]. When there was a gap of more than 7 days between prescription refills, patients were considered to have discontinued the medication. The presence of other medications at discharge was also assessed from VA external peer review program (EPRP) chart review. Glomerular filtration rate (eGFR, ml/min/1.73 m^2^) was calculated by the four-variable CKD-EPI estimating equation, and patients were classified by strata of eGFR consistent with current guidelines [34]. Other comorbid health conditions during hospitalization were identified by ICD-9CM codes. Data regarding PCI, stent placement and type, and thrombolysis in myocardial infarction (TIMI) score were also recorded.

### Outcomes and data sources

The primary outcomes were: i) all-cause mortality ii) readmission/hospitalization for AMI, iii) a combined measure of all-cause mortality or readmission/hospitalization for AMI, and iv) major bleeding. Death was ascertained in the VA Vital Status File [35,36]. The re-admission/re-hospitalization AMI outcome was based on a primary discharge *International Classification of Diseases, Ninth Revision, Clinical Modification* diagnosis code of 410.XX for any hospitalization within the VA. Major bleeding events were defined by either i) a hospitalization with a primary ICD-9 CM code for bleeding [430.xx (subarachnoid hemorrhage), 431.xx (intracerebral hemorrhage), 432.xx (other and unspecified intracranial hemorrhage), 578.xx (gastrointestinal hemorrhage) 719.1x (hemarthrosis), 423.0x (hemopericardium), 599.7x (hematuria), 626.2x (excessive or frequent menstruation), 626.6x (metrorrhagia), 626.8x (other gynecologic bleeding), 627.0x (premenopausal menorrhagia), 627.1x (postmenopausal bleeding), 786.3x (hemoptysis), 784.7x (epistaxis), 459.0x (hemorrhage NOS)] OR ii) a secondary ICD-9 CM code for bleeding and blood transfusion (99.0x).

### Statistical analyses

Patient characteristics at entry into the cohort were described overall and by eGFR strata using mean +/− standard deviation (SD) for quantitative variables and frequencies and percents for categorical variables. Bivariate analyses involving ANOVA and Chi-square tests were used as appropriate to assess differences in patient characteristics.

Corresponding Kaplan-Meier curves were constructed to display the cumulative incidence of these four events. In computations of event rates and in Cox regression analyses, follow-up time was censored at discontinuation of clopidogrel use or lost to follow up. All cause death was ascertained until the administrative end date of the study.

Cox proportional hazards regression models were used to assess the association between each of these four outcomes and eGFR strata (defined at hospital discharge) among all participants who received clopidogrel at hospital discharge. The assumption of proportional hazards in the Cox regression models was checked using Schoenfeld residuals for all included covariates. Variables that did not meet the assumptions where stratified before including them in the model.

All analyses were conducted using Stata version 9.0 (StataCorp, College Station, Texas). The Cardiac Care Follow-up Clinical Study was approved by the University of Washington’s human subjects committee and VA Institutional Review Board.

## Results

### Study sample and characteristics

Among the 7413 individuals in the final analytic cohort, the majority was male (98%), white (80%), and elderly (mean age 66 years) (Table 1). The cohort was distributed by eGFR levels as follows: 53.9% with eGFR > = 60 1.73 ml/min/m^2^, 34.5% with eGFR 30–60 1.73 ml/min/m^2^, and 11.6% with eGFR < 30 1.73 ml/min/m^2^. A large proportion of the cohort had chronic medical conditions, especially hyperlipidemia (76%), diabetes (42%), and hypertension (28%). Fifty-nine percent of the cohort had a TIMI score > = 3 and 22% had a left ventricular ejection fraction (LVEF) < 40%. While 42% did not have a percutaneous coronary intervention (PCI) during hospitalization, 45% had a PCI with a drug eluting stent (DES) and 13% had a PCI with a bare metal stent (BMS).

**Table 1:** Characteristics among patients discharged on clopidogrel at hospital discharge by eGFR strata

	**Overall (n = 7413)**	**eGFR > = 60 ml/min/m2 (n = 3997)**	**eGFR 30–60 ml/min/m2 (n = 2559)**	**eGFR < 30 ml/min/m2 (n = 857)**	**p-value**
Age (mean +/− SD)	66.3 (11.2)	61.9 (9.9)	71.2 (10.5)	71.9 (10.5)	<0.001
Male sex (%, n)	98 (7294)	98 (3936)	98 (2513)	99 (845)	0.61
White race (%, n)	80 (5963)	82 (3269)	81 (2083)	71 (611)	<0.001
Medical conditions (%, n)					
Diabetes	42 (3139)	35 (1385)	47 (1209)	64 (545)	<0.001
Hypertension	28 (2101)	21 (843)	33 (840)	49 (418)	<0.001
H/o MI	19 (1410)	17 (692)	21 (544)	20 (174)	<0.001
CHF	13 (999)	6 (227)	18 (453)	37 (319)	<0.001
Stroke	2 (172)	2 (65)	3 (75)	4 (32)	<0.001
PAD	13 (974)	10 (398)	15 (395)	21 (181)	<0.001
Lipid disorder	76 (5627)	77 (3070)	77 (1976)	68 (581)	<0.001
Liver disease	2 (177)	3 (108)	2 (50)	2 (19)	0.15
COPD	12 (902)	11 (439)	14 (355)	13 (108)	.002
Cancer	5 (388)	5 (182)	6 (155)	6 (51)	.017
Dementia	4 (309)	4 (152)	5 (119)	4 (38)	0.226
Depression	9 (642)	10 (399)	8 (196)	5 (47)	<0.001
Current smoker (%, n)	31 (2333)	42 (1681)	21 (537)	13 (115)	<0.001
LVEF (%, n)					<0.001
EF > = 40%	73 (5430)	78 (3121)	70 (1786)	61 (523)	
EF < 40%	22 (1622)	16 (630)	27 (688)	35 (304)	
missing	5 (361)	6 (246)	3 (85)	4 (30)	
Procedure (%, n)					<0.001
No PCI	41 (2990)	31 (1215)	47 (1208)	66 (567)	
PCI/no stent	2 (175)	3 (109)	2 (47)	2 (19)	
PCI/BMS	13 (929)	14 (540)	12 (311)	9 (78)	
PCI/DES	45 (3287)	53 (2111)	39 (984)	22 (192)	
TIMI > = 3 (%, n)	59 (4371)	48 (1901)	71 (1821)	76 (649)	<0.001
Glycoprotein inhibitor (%, n)	46 (3394)	53 (2130)	41 (1059)	24 (205)	
Hospital discharge medications (%, n)					
ASA	95 (7016)	96 (3821)	94 (2404)	92 (791)	<0.001
Beta-blocker	93 (6925)	94 (3752)	93 (2375)	93 (798)	0.223
ACE-I	71 (5276)	77 (3068)	70 (1799)	48 (409)	<0.001
ARB	9 (644)	6 (256)	11 (280)	13 (108)	<0.001
heparin	4 (264)	4 (142)	4 (100)	3 (22)	0.186

**Table 2:** Days of clopidogrel use during 1 year after hospital discharge by presence of PCI and eGFR strata

	**No PCI**				**PCI**			
	**N**	**Mean days**	**SD**	p-value*	**N**	**Mean days**	**SD**	p-value*
**eGFR level**				<0.01				<0.01
> = 60	1215	241	130		2782	297	105	
30-60	1208	232	132		1351	293	110	
<30	567	209	130		290	259	126	

*comparison among three levels of eGFR.

A progressive increase in mean age and frequency of non-white race was observed with lower strata of eGFR (p < 0.001). The majority of comorbid conditions (e.g., diabetes, hypertension, stroke, peripheral vascular disease, and history of myocardial infarction) were significantly more prevalent among individuals with lower eGFR strata <60 than in those with higher eGFR > =60 (p < 0.001). While beta-blocker use at discharge was similar across strata of eGFR, ACE-I use was significantly less frequent in those with lower eGFR (<60) (p < 0.001). LVEF < 40% and a TIMI score > = 3 was progressively greater in individuals with lower eGFR (p < 0.001). The proportion of individuals receiving PCI steadily decreased with lower eGFR (69% with eGFR > 60; 34% with eGFR < 30) (p < 0.001). Similarly, PCI with DES, and PCI with BMS was progressively lower in those with lower eGFR (p < 0.001).

### Duration of clopidogrel use

Duration of clopidogrel use following hospital discharge was progressively shorter with lower eGFR level among patients with or without PCI (p < 0.01) Table 2. Among those without PCI, mean (SD) days of clopidogrel use was 241 (130) in individuals with eGFR > =60 and 209 (130) in individuals with eGFR < 30, while among those with PCI, mean (SD) days of clopidogrel use was 297 (105) and 259 (126) in those with eGFR > =60 and < 30, respectively.

### Clinical outcomes by eGFR strata

**Figure 1:**
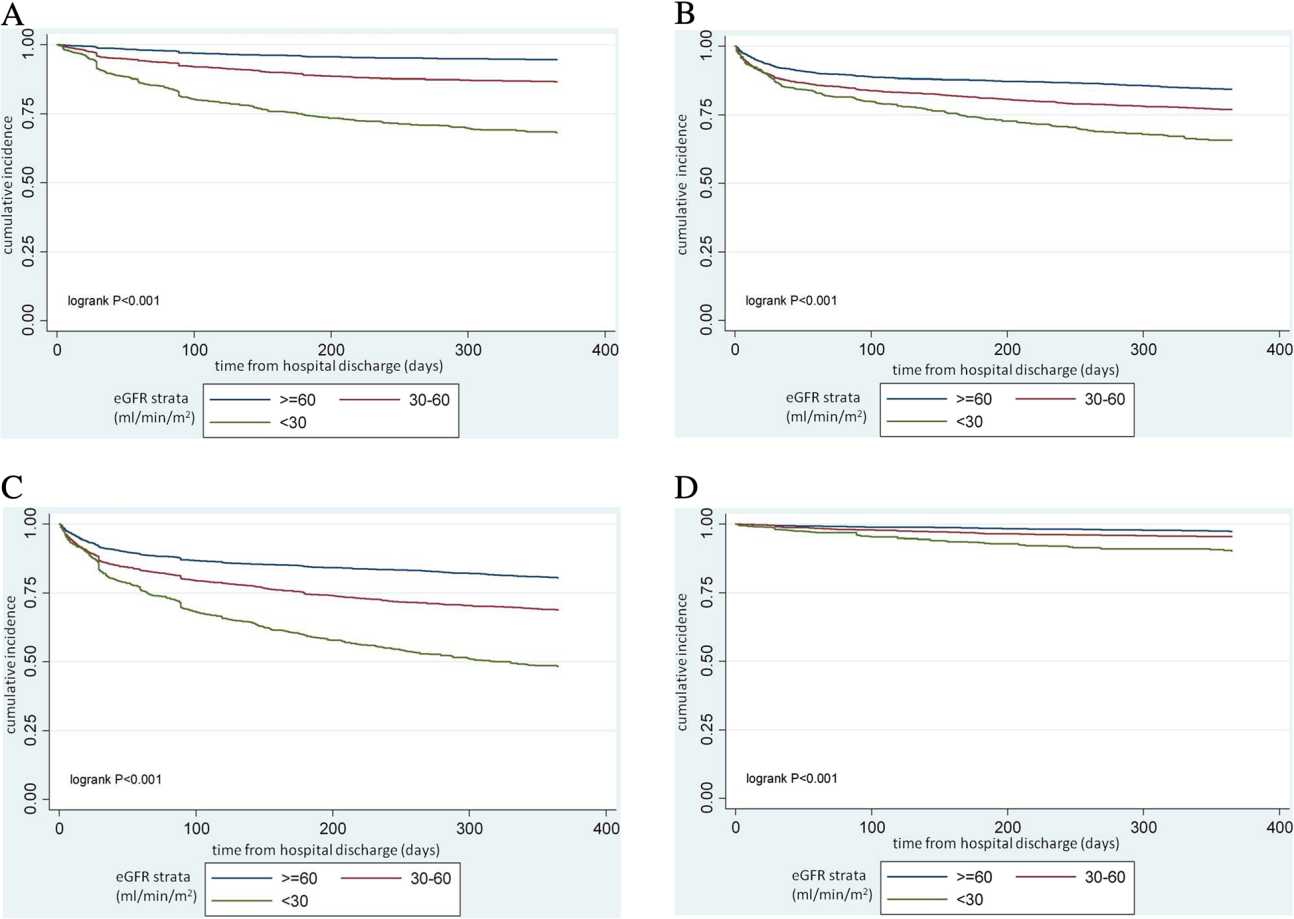
**Cumulative Incidence of Outcomes by eGFR Strata (ml/min/1.73m2). A**: Cumulative Incidence of All-cause Death by eGFR Strata (ml/min/1.73 m^2^). **B**: Cumulative Incidence of Readmission/Hospitalization for Acute Myocardial Infarction by eGFR Strata (ml/min/1.73 m^2^). **C**: Cumulative Incidence of All-cause Death or Readmission/Hospitalization for Acute Myocardial Infarction by eGFR Strata (ml/min/1.73 m^2^). **D**: Cumulative Incidence of Major Bleeding by eGFR Strata (ml/min/1.73 m^2^).

During 1 year of follow up after hospital discharge, 10% of the cohort died, 18% had a hospitalization for AMI, 25% either died or had a hospitalization for AMI, and 4% had a major bleeding event. The cumulative incidence of each of these outcomes was progressively higher for lower strata of eGFR (Figure 1A-D). While 5% of patients with an eGFR > =60 died, 12% of those with an eGFR 30–60 and 28% of those with an eGFR < 30 died during follow up (p < 0.001). Similarly, only 14% of individuals with an eGFR > = 60 had a hospitalization for AMI, which contrasted with the 21% of those with an eGFR 30–60 and 27% with an eGFR < 30 who required hospitalization for AMI (p < 0.001). Lastly, although a major bleeding event occurred among 3% of patients with an eGFR > = 60, such events happened in 5% of those with an eGFR 30–60 and 10% of those with an eGFR < 30 (p < .001).

### Association between eGFR and clinical outcomes

In regression analyses adjusted for several demographic characteristics, medications, and clinical factors, low eGFR was significantly associated with an increased risk for each of the outcome measures (Table 3). Compared to those with an eGFR > =60, individuals with an eGFR 30–60 and < 30 had nearly 1.5 fold higher (HR 1.45; 95% CI: 1.18-1.76) and 2.5 fold higher (HR 2.48; 95% CI: 1.97-3.13) risk of death, respectively. Similarly, a progressive increased risk of hospitalization for AMI was associated with declining eGFR, HR 1.20; 95% CI: 1.04-1.37 and HR 1.47; 95% CI: 1.22-1.78 for eGFR 30–60 and < 30, respectively. Also, an increased risk for the composite outcome of death or hospitalization for AMI was observed with eGFR 30–60 (HR 1.22; 95% CI: 1.08-1.37) and eGFR < 30 (HR 1.77; 95% CI: 1.52-2.06) compared with an eGFR > = 60. Compared with the highest strata eGFR (> = 60), while eGFR 30–60 was not associated with a significant increased risk of major bleeding (HR 1.10; 95% CI: 0.80-1.53), the lowest strata of eGFR (<30) was independently associated with over a 2 fold increased risk in major bleeding (HR 2.09; 95% CI: 1.40-3.12).

**Table 3:** Association between eGFR and clinical outcomes among patients using clopidogrel*

**eGFR strata**	**Adjusted HR (95% CI)**
All cause mortality	
> = 60	1.0 (ref)
60 - 30	1.45 (1.18-1.76)
< 30	2.48 (1.97-3.13)
Readmission/hospitalization for AMI	
> = 60	1.0 (ref)
60 - 30	1.20 (1.04-1.37)
< 30	1.47 (1.22-1.78)
All cause mortality or readmission/hospitalization for AMI	
> = 60	1.0 (ref)
60 - 30	1.22 (1.08-1.37)
< 30	1.77 (1.52-2.06)
Major bleeding	
> = 60	1.0 (ref)
60 - 30	1.10 (0.80-1.53)
< 30	2.09 (1.40-3.12)

*adjusted for age, sex, race, comorbid conditions (Table 1), smoking, medications (Table 1), PCI/stent, LVEF, TIMI score.

## Discussion

In a large national cohort of elderly patients hospitalized with ACS and prescribed clopidogrel at discharge, moderate and severe CKD were associated with a greater risk of several unfavorable clinical outcomes and complications. A graded increased risk of all cause mortality and hospitalization for AMI was observed with lower levels of eGFR, including nearly a 2.5- and 1.5-fold increased risk with eGFR < 30, respectively. Over a 2-fold increased risk of major bleeding events was associated with eGFR < 30. In addition, a progressively shorter duration of outpatient clopidogrel therapy after hospital discharge was observed with lower levels of eGFR, regardless of whether PCI was performed. Among patients with PCI, patients with eGFR < 30 had taken clopidogrel 13% fewer days than those with eGFR > = 60 during follow up.

Several studies of ACS have demonstrated a negative relationship between CKD, recurrent MI, and survival [3-10]. Two post-hoc analyses of clinical trials comparing clopidogrel to placebo found that CKD (eGFR < 60) was associated with an increased risk of a composite outcome MI, CV death or stroke [17,18]. Observational studies of clopidogrel use post PCI have noted higher rates of stent restenosis, myocardial infarction and death in patients with low kidney function compared to those with normal kidney function [26-28]. However, these studies have important limitations such as including few patients with severe CKD, unaccounting of clopidogrel treatment duration, comprising single center cohorts, utilizing inferior methods of estimating kidney function, and failing to include patients without PCI [26-28].

In contrast to these prior reports, we included both patients with and without PCI, and robustly examined patients with very low eGFR (< 30), finding them to be at exceptionally high risk for adverse outcomes. Multiple reasons likely underlie the marked association between severe CKD and poor outcomes, including a greater number of cardiovascular risk factors, more complex coronary anatomy, increased overall atherosclerotic disease burden, and lower frequency of PCI and other recommended ACS treatments during hospitalization and follow up in patients with CKD [26-28]. Patients with CKD have been shown to have a greater risk of thrombotic complications including stent thrombosis [6,37], and it has been posited that a prothrombotic state may exist in CKD as evidenced by increases in fibrinogen, vonWillebrand’s factors, and decreases in antithrombin III [22,23].

Many types of cardioprotective medications and other evidence-based treatments are underutilized in adults with CKD and cardiac risk factors or ACS [38-42]. Similar to our observations of a shorter duration of clopidogrel use in patients with lower eGFR after ACS, a multicenter study of patients undergoing PCI and stent placement noted that as creatinine clearance declined, there was a decrease in the percent of patients taking clopidogrel at 1 year post-procedure [28]. A one year prospective study of 1622 adults prescribed antiplatelet therapy after drug-eluting stent placement observed that 14% interrupted at least one antiplatelet drug during this time, including 11.8% who stopped clopidogrel [43]. Renal impairment was associated with nearly a 3 fold increased odds of discontinuation of antiplatelet drugs [43]. Reasons for shorter duration of antiplatelet therapy and discontinuation observed in these studies and ours may include bleeding events, scheduled invasive procedures, psychiatric drug use, unemployment, patient choice and non-adherence, and other medical events not specified including earlier mortality [43].

Concern for antiplatelet therapy-related bleeding events may curtail provider treatment of patients with CKD following ACS with these medications [28]. Others have observed a progressive increase in both minor and major bleeding events with lower levels of kidney function for patients during hospitalization for ACS [28] or in the course of follow up thereafter while on antiplatelet therapies [17,18,27]. Fewer have demonstrated an independent relationship between low levels of kidney function and bleeding. While a prior study found an independent relationship between creatinine clearance (CrCl) < 30 ml/min and in-hospital bleeding events [28], we observed a strong independent relationship between eGFR < 30 and an increased risk of major bleeding events after hospital discharge. Platelets are known to be dysfunctional in the setting of CKD because of intrinsic platelet abnormalities and impaired platelet-vessel wall interactions, which may account for the increased risk of bleeding in patients with CKD [18-23,44].

While this study leveraged a large national cohort with varying degrees of CKD, meticulous data collection, and outcome ascertainment, it does have limitations. First, because this study has an observational study design, it is subject to residual confounding and its results do not indicate causality [45]. Furthermore, because this study lacked a comparison group, it is not possible to make conclusions regarding the risks and benefits of using clopidogrel versus no clopidogrel in the CKD population from this study. Nevertheless, it should be noted that longitudinal studies are appropriate to robustly assess epidemiologic relationships [45]. Second, because this cohort consists exclusively of patients hospitalized at VA facilities, its findings may not be generalizable to populations elsewhere. However, elderly male Veterans are a vulnerable high-risk group that is subject to health disparities and critically important to study [46]. Third, we calculated eGFR and classified CKD based on a single outpatient serum creatinine value. Similarly, we assigned comorbid conditions based on a single ICD-9 code being present in administrative data sources. Although this approach is acceptable in clinical and research settings, some misclassification with comorbidity assignment and CKD staging is possible. Fourth, we did not have data regarding clopidogrel dose, but it does not appear to significantly impact clinical outcomes [47]. Similarly, while we employed meticulous medication data collection throughout hospitalization and follow up, there may be medications not accounted for that may alter the efficacy and risk profile of clopidogrel [48-50].

## Conclusions

In summary, among patients hospitalized with ACS and treated with clopidogrel, lower levels of kidney function were associated with a greater risk of death, hospitalization for AMI, and major bleeding. These findings should remind clinicians of the increased risks for adverse outcomes in their CKD patients following ACS who are treated with clopidogrel. Further studies are needed in order to provide sound evidence-based decision making for management decisions in this high-risk group following ACS.

## Abbreviations

CV: Cardiovascular; CKD: Chronic kidney disease; ACS: Acute coronary syndrome; PCI: Percutaneous coronary intervention; VA: Department of Veterans Affairs; AMI: Acute myocardial infarction; UA: Unstable angina; ICD-9CM: International Classification of Diseases, Ninth Revision, Clinical Modification; EPRP: External peer review program; eGFR: Estimated glomerular filtration rate; TIMI: Thrombolysis in myocardial infarction; SD: Standard deviation; LVEF: Left ventricular ejection fraction; DES: Drug eluting stent; BMS: Bare metal stent; HR: Hazard ratio; CrCl: Creatinine clearance.

## Competing interests

None of the authors has financial or non-financial competing interest to disclose.

## Authors’ contributions

MJF: made substantial contributions to conception and design, or acquisition of data, or analysis and interpretation of data; has been involved in drafting the manuscript or revising it critically for important intellectual content; and has given final approval of the version to be published. PMH: made substantial contributions to conception and design, or acquisition of data, or analysis and interpretation of data; has been involved in drafting the manuscript or revising it critically for important intellectual content; and has given final approval of the version to be published. KM: made substantial contributions to conception and design, or acquisition of data, or analysis and interpretation of data; and has given final approval of the version to be published. EL: made substantial contributions to conception and design, or acquisition of data, or analysis and interpretation of data; and has given final approval of the version to be published. CRP: made substantial contributions to conception and design, or acquisition of data, or analysis and interpretation of data; has been involved in drafting the manuscript or revising it critically for important intellectual content; and has given final approval of the version to be published. All authors read and approved the final manuscript.

## Pre-publication history

The pre-publication history for this paper can be accessed here:

http://www.biomedcentral.com/1471-2369/14/107/prepub
